# Supplementary figures and images for: A close unicellular animal relative and predator of schistosomes exhibits chemokinesis in response to proteins and peptides from its prey
Source: PLoS Pathog. 2025 Sep 3;21(9):e1013440. doi: 10.1371/journal.ppat.1013440 (PMC12422584; doi:10.1371/journal.ppat.1013440)

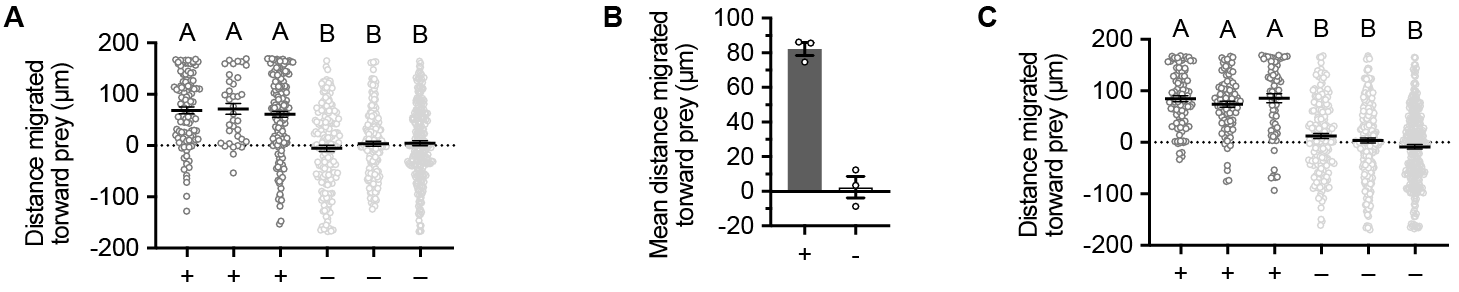

Supplement: S1 Fig — (A) Migration of individual cells toward schistosome prey (same data that is summarized in Fig 1B). The three left samples have a schistosome present. The three right samples do not (‘prey’ location was a randomly chosen spot in the middle of the field of view). Lines indicate means, and error bars represent standard error of the mean. Each circle is a single tracked cell. One-way ANOVA with multiple comparisons was performed, showing that all samples with a schistosome had significantly higher motility toward the schistosome than all three samples lacking a true schistosome. (B–C) Repeated chemotaxis experiment using fibronectin-coated dishes instead of plain tissue culture-treated surfaces (parallel to Figs 1B and S1A). (B) Chemotaxis of Capsaspora toward schistosome sporocysts. Average net movement toward the schistosome is reported (negative value would indicate movement away from schistosome). Error bars represent standard error of the mean of a biological triplicate of three individual wells of cells (n = 3). Individual biological replicates are displayed with white circles. Within each biological replicate, many individual cells were tracked. (C) Migration of individual cells toward schistosome prey (same data that is summarized in S1B Fig). The three left samples have a schistosome present. The three right samples do not (‘prey’ location was a randomly chosen spot in the middle of the field of view). Lines indicate means, and error bars represent standard error of the mean. Each circle is a single tracked cell. One-way ANOVA with multiple comparisons was performed, showing that all samples with a schistosome had significantly higher motility toward the schistosome than all three samples lacking a true schistosome. (TIF) [file ppat.1013440.s001.tif]

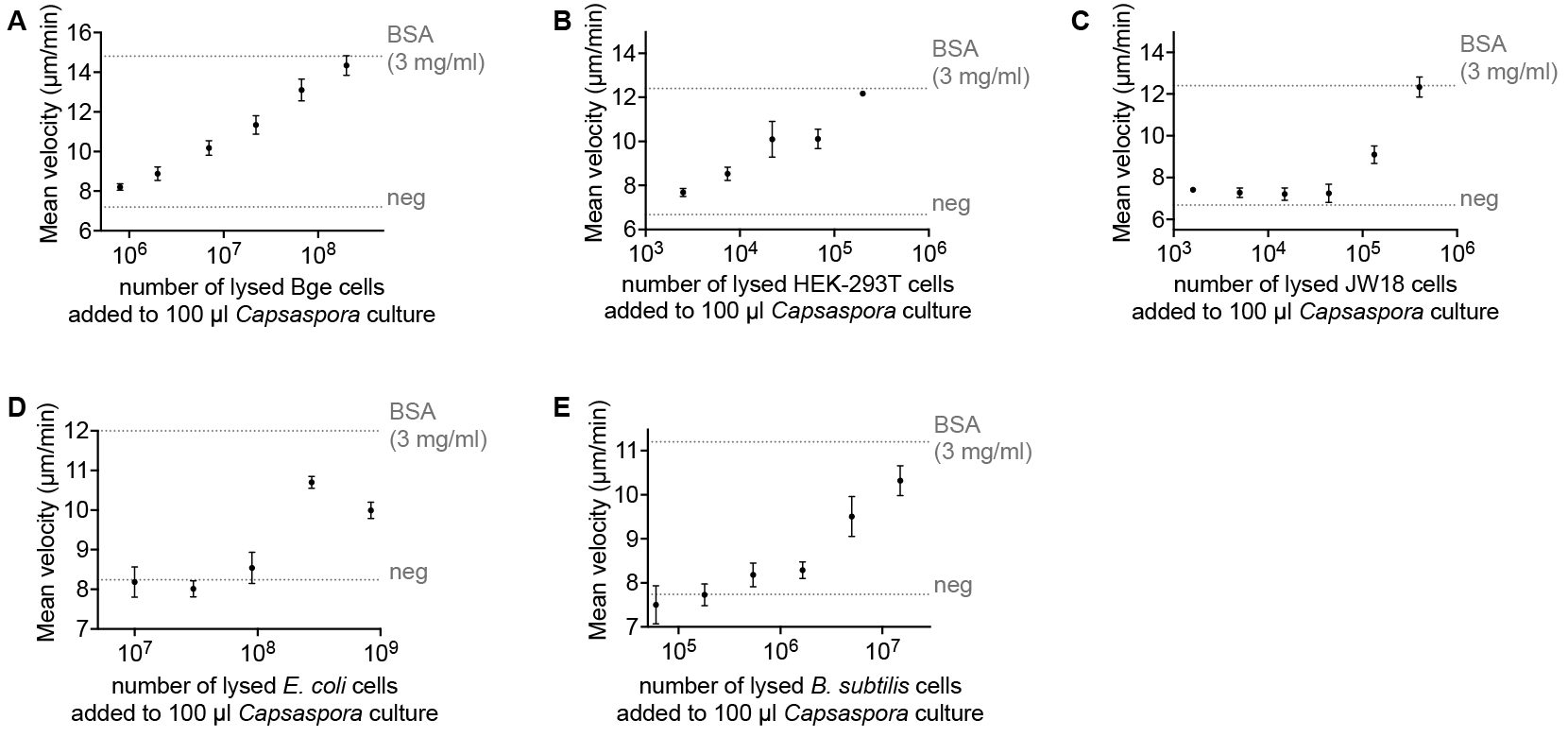

Supplement: S2 Fig — Data match those plotted in Fig 1C–1G, where the data from only the most active concentration was plotted for each lysate. In all cases except for E. coli lysate, this was the highest concentration tested. (A) Capsaspora motility upon addition of different concentrations of Bge cell lysate. (B) Capsaspora motility upon addition of different concentrations of HEK-293T cell lysate. (C) Capsaspora motility upon addition of different concentrations of JW18 cell lysate. (D) Capsaspora motility upon addition of different concentrations of E. coli cell lysate. (E) Capsaspora motility upon addition of different concentrations of B. subtilis cell lysate. In all cases, the final concentration of lysed cell material added in the cell culture is displayed on the x-axis. Error bars represent standard error of the mean of a biological triplicate of three individual wells, each with dozens of cells (n = 3). The dashed lines indicate baseline motility upon addition of negative control (water) or induced motility upon addition of 3 mg/ml BSA. (TIF) [file ppat.1013440.s002.tif]

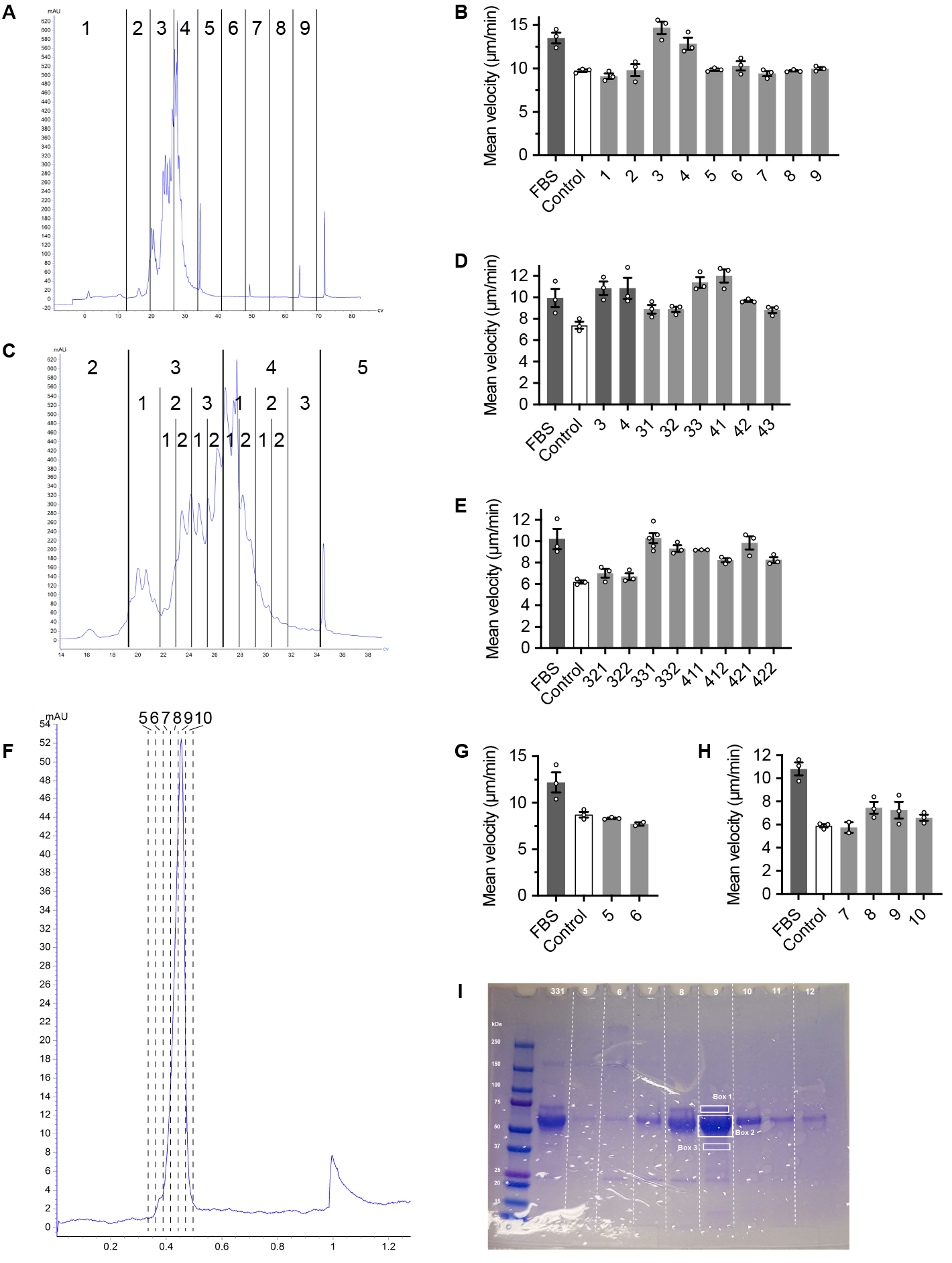

Supplement: S3 Fig — (A) Chromatogram of FBS separated by anion exchange (AEX) chromatography. Coarse pooled fractions are shown. (B) Capsaspora motility upon addition of coarse fractions from AEX. (C) Chromatogram of FBS separated by anion exchange (AEX) chromatography. Medium-sized and fine-sized pooled fractions are shown. (D) Capsaspora motility upon addition of medium-sized pooled fractions from AEX. (E) Capsaspora motility upon addition of fine-sized pooled fractions from AEX. (F) Chromatogram of FBS separated by size exclusion chromatography (SEC). Fractions collected are shown. (G) Capsaspora motility upon addition of fractions 5 and 6 from SEC. (H) Capsaspora motility upon addition of fractions 7–10 from SEC. (I) Coomassie-stained SDS-PAGE gel of SEC fractions. Boxes are placed around gel bands that were excised for identification by tryptic peptide LC-MS/MS. For all motility assays, positive control (10 µl whole FBS) and negative control (10 µl CBSS) were included on the same day as the co-plotted sampled. In all plots, error bars represent standard error of the mean of a biological triplicate (in a couple cases duplicate) of individual wells, each with dozens of cells (n = 3). Individual replicates are displayed with white circles. (TIF) [file ppat.1013440.s003.tif]

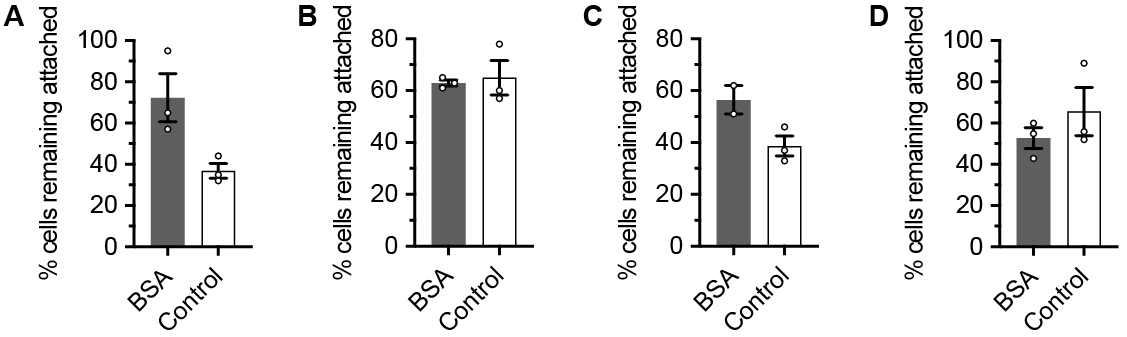

Supplement: S4 Fig — Capsaspora adhesion to tissue culture-treated plastic microplate wells upon addition of 3 mg/ml FBS. Each panel is an experiment run on a separate day. The mean from each experiment was used to generate Fig 4A. Error bars represent standard error of the mean of two or three individual wells, each containing dozens of cells. Individual biological replicates are displayed with white circles. (TIF) [file ppat.1013440.s004.tif]

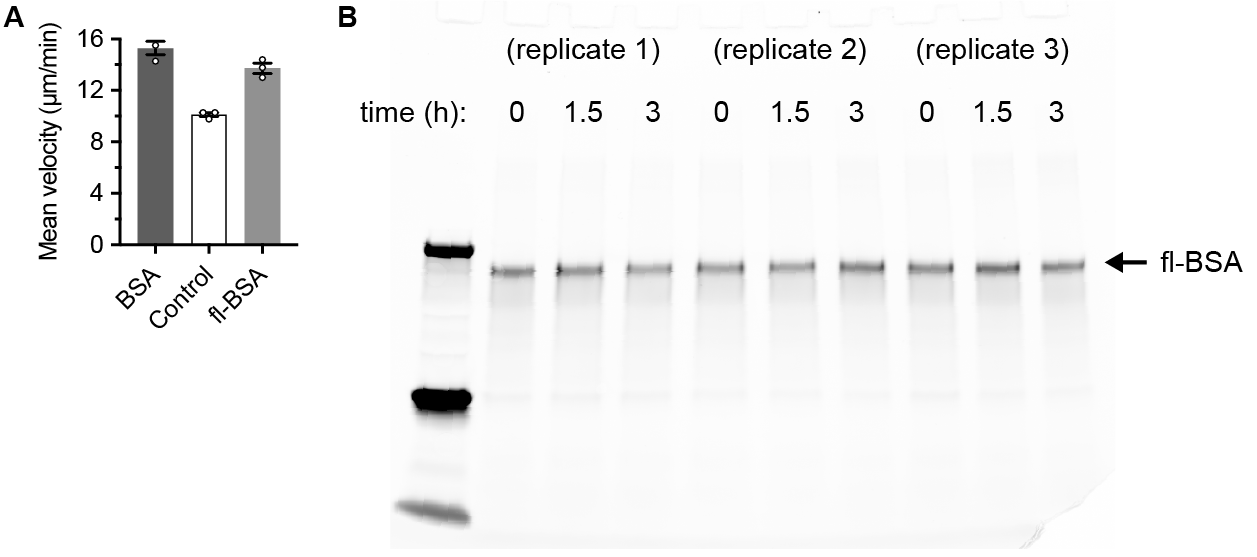

Supplement: S5 Fig — (A) Capsaspora motility upon addition of 3 mg/ml of BSA or fluorescently modified BSA (fl-BSA), compared to a negative control (water). Error bars represent standard error of the mean of a biological triplicate of three individual wells, each with dozens of cells (n = 3). Individual biological replicates are displayed with white circles. (B) Fluorescence-scanned SDS-PAGE gel used to quantify the remaining fluorescent BSA after incubation with Capsaspora. The fl-BSA bands were quantified to generate Fig 4C. (TIF) [file ppat.1013440.s005.tif]

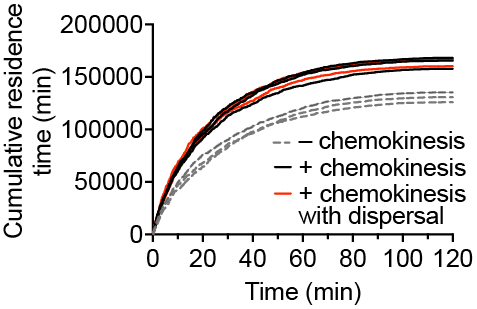

Supplement: S6 Fig — Cumulative residence times (i.e., the total sum of time that the 2000 Capsaspora cells are attached to the schistosome over the 120 minute simulation) with and without chemokinesis over time. Three replicate simulations for each condition are shown as individual curves. Grey dashed curves show the non-chemokinesis condition. Black solid curves show the chemokinesis condition. Red solid curves represents the case where chemokinesis is enabled and four nearby schistosomes were depleted and gave a final burst of chemo-effector at the start of the simulation—no significant difference was observed compared to the standard chemokinesis condition. Fig 5C summarizes the cumulative residence times at the endpoint (120 minutes). (TIF) [file ppat.1013440.s006.tif]
