# Supplementary material for: A close unicellular animal relative and predator of schistosomes exhibits chemokinesis in response to proteins and peptides from its prey
Source: PLoS Pathog. 2025 Sep 3;21(9):e1013440. doi: 10.1371/journal.ppat.1013440 (PMC12422584; doi:10.1371/journal.ppat.1013440)
Supplement: Attachment — Submitted filename: chemokinesis response letter3.pdf [file ppat.1013440.s013.pdf]

INDIANA UNIVERSITY

July 28, 2025

Dr. Dominique Soldati-Favre
PLOS Pathogens
plospathogens@plos.org

Dear Dr. Soldati-Favre,

Enclosed please find our revised manuscript entitled, "A close unicellular animal relative and predator of schistosomes exhibits chemokinesis in response to proteins and peptides from its prey" (PPATHOGENS-D-25-01241) that we are submitting for consideration in *PLOS Pathogens*.

We were delighted to learn that both Reviewers found the work interesting. As requested, we have revised the manuscript considering the Editor and Reviewers' comments. Our point-by-point responses to the queries and our changes to the text are provided in detail below:

Editor:

1. The Editor states: *'The authors need to clarify and correct inconsistencies in data presentation, video labeling, and interpretation, particularly regarding velocity comparisons, stimulus specificity, and the coherence of the mathematical model.'*

We thank the editor and have relabeled the videos, both in the manuscript and in the new deposition on IU Data Core. We have further discussed the differences in experimental setups that caused the different velocities [lines 151-155], added discussion about the promiscuity of the stimulus [lines 808-824], and put the mathematical model into better context [lines 417-432].

2. The Editor states: '*They should also elaborate on the biological relevance of Capsaspora's chemokinetic response, including comparisons between schistosome material and generic stimuli like BSA, and discuss potential in vivo implications.'*

We thank the editor and have added discussion text comparing these stimuli and speculating on the potential in vivo implications [lines 808-824].

3. The Editor states: '*Additionally, the manuscript would benefit from clearer explanations of experimental design, key concepts such as aggregation versus chemokinesis, residence time, and collective behavior, as well as improved consistency in terminology, figure captions, and acronym usage.'*

We thank the editor and have added new text in the experimental section and figure captions to clarify experimental design, including how we avoiding confounding results from aggregation. We have also added text in the results clarifying the term "residence time" [lines 428-432] and referencing prior work that initial *Capsaspora* cells engage with the prey through random movement [line 163]. Finally, we have defined the 'HSA' acronym at its first use in the text [lines 262 & 285].

800 E. Kirkwood Ave., Room A563
Bloomington, IN 47405
Department of Chemistry
812/855-2068
jpgerdt@iu.edu

Reviewer 1:

All “minor issues”:

1. The Reviewer states: *‘I felt that some of the biological relevance was lost when it was noted that Capsaspora is attracted both to 'leaking' Schistosomes, various cell lysates and BSA. In the internal environment of a snail, there could presumably be any number of competing chemical gradients. Could the authors discuss any evidence that the attraction to material from schistosomes is specific/stronger than that to BSA, and whether future experiments to analyze the biological relevance of the effect might be worthwhile, and if so, which ones?’*

We thank the reviewer and have added further discussion about the potential benefits and challenges afforded by responding to a general cue (lines 808-824 of marked manuscript).

2. The Reviewer states: *‘the abstract mentions that Capsaspora could be a model for the unicellular ancestor of animals. This aspect doesn't seem to be picked up in the discussion or conclusion; can the authors elaborate on this?’*

We thank the reviewer for pointing this out. We have added text to the discussion and conclusion that more explicitly discuss the implications of our work with regards to *Capsaspora* as an evolutionary model organism (lines 856-868 and 893-897).

3. The Reviewer states: *‘The collective behavior is interesting; can the authors confirm that the initial attachment of one Capsaspora cell is random? I think that they say this at some point but couldn't find mention in the section entitled "Chemokinesis response is cell-density-dependent but not contact-dependent", which seems like a relevant place.*

That is correct based on the initial observation in the literature. We have added an explicit mention of this with the reference (line 163 of marked manuscript).

4. The Reviewer states: *‘There's a statement on p.17 of the review document (paragraph beginning "previous mathematical models [...]") that chemokinesis doesn't alter the trajectories, only the speed at which they're traversed (or words to that effect), which I agree with. I didn't quite understand the following statement that 'the number of cells that reach the schistosome are unchanged by the speed of locomotion', especially in light of the last sentence of that paragraph about arrival rates increasing in the case of irreversible attachment. Can the authors clarify?’*

We thank the reviewer for this comment and have rewritten the paragraph to clarify that cells will reach the prey faster and therefore reside on the prey longer [lines 417-432].

5. The Reviewer states: ‘*Following from this, can the authors clarify what they mean by 'residence times' of the Capsaspora cells at the schistosome in the main document? It's discussed in the SI, but it's quite important to define it in the main body too. The same goes for the differences in the simulation of HSA and BSA, as it's not clear what these will mean in practice from the main text.’*

We thank the reviewer and have added text to define “residence times” in the main text [lines 428-432]. We have removed the HSA data because it showed no meaningful different with BSA in a test simulation with the new conditions.

6. The Reviewer states: *‘On a related note, can the authors clarify the following sentence: "We considered the possibility that the long-term attachment of Capsaspora to prey may be sufficient to*

make chemokinesis a beneficial trait; however, we found that to be untrue." Can they point to which experiment/simulation shows this, or was it not tested directly?'

This section is changed in our revision. Following the advice of Reviewer 2, we adjusted several factors of our simulation, which now showed an advantage to chemokinesis. Our revised text (lines 433-749) clearly discusses how the new model quantified a benefit for chemokinesis.

7. The Reviewer states: *'Typos/presentational issues:*
a) Fig. 1B describes the mean distance migrated towards/away from a schistosome. Can the authors include in the caption how long the cells were monitored for? The details are probably in the methods somewhere, but the information is useful here.

As recommended, the bold text was added: "Data were derived from Videos S1-S3 (with schistosome) and Videos S4-S6 (without schistosome) **tracking cells for 4 hours. Only cells that were tracked for ≥ 2 hours were included in the analysis.**" This detail was also added to the methods (lines 979-980 in the marked manuscript).

b) Along similar lines, the caption to Fig. 1B mentions that only Capsaspora cells 70-240um from the schistosome were tracked. Can the authors explain why? This might be due to something straightforward, e.g. the microscope's fixed field of view.

As recommended, the bold text was added: "Only Capsaspora cells 70 μm to 240 μm from the schistosome were tracked **because cells closer than 70 μm might attach to the schistosome via filopodia, and cells further than 240 μm were near the boundaries of the field of view and may be near other schistosomes.**" This detail was also added to the methods (lines 972-974 in the marked manuscript).

c) 4-5 lines above Fig. 3: should this read "They slightly increased motility at a single [...]"? i.e. 'increased' rather than 'induced'?

We thank the reviewer for the suggestion and have completed it.

d) Could the authors explain the acronym 'HSA' at first use in the main document?'

We thank the reviewer for the suggestion and have completed it. (lines 262 & 285 of marked manuscript)

Reviewer 2:

“major issues”

1. The Reviewer states: *‘Figure 1. Interestingly the mean value of velocity when exposed to schistosome (c), is around 6 microm/minute which is very similar to the mean velocity that appears to have the Capsaspora control in D/E/F and G. Does this mean that schistosomes are the less powerful trigger from all the ones assayed? Or mean velocities vary a lot from experiment to experiment and are not comparable? It would be nice that the authors elaborate a bit on this.’*

We thank the reviewer for their careful reading! We believe these values are an artifact of the different experimental setups. It is analogous to the ‘coastline paradox’. In the case of the schistosome, each image was 1 minute apart. In later analyses, each image was 5 seconds apart. In both cases, data were reported as μm/min, but in the later analysis, the 20x more readings revealed many more subtle movements within that minute leading to a larger total distance measured compared to the original video, which only gave a single displacement value for each minute. We added the following text to the end of the Figure 1 caption: “**We note that movement appears faster in assay method B compared to the case of schistosome co-culture (panel C). This is believed to be an artifact of 20× more frequent measurements in method B reporting a truer full distance moved per minute, compared to the one-frame-per-minute displacement values from the co-culture experiment.**”

2. The Reviewer states: *‘Videos S1 and S3 correspond to Capsaspora with schistosome and S4 to S6 without schistosome, so reference in the text (figure Legend 1 and first paragraph of results) is wrong. Also difficult to make sense with the legend in the Fig Share where states that all videos had schistosome, but different treatment of the flasks, but in the manuscript only talks about the presence/absence of schistosome. Please unify and clarify. FIG SHARE: “ Videos of Capsaspora responding to schistosome sporocyst on plain tissue culture-treated plastic surface (Videos 1-3) and on fibronectin-treated surface (Videos 4-6).”’*

We thank the reviewer for pointing out the discrepancy. We have re-labeled the Figure 1 caption (S1-S3 has schistosome & S4-S6 do not), and we have re-labeled the caption—now on IU Data Core (“Capsaspora increases motility in response to cell lysates and pure proteins and peptides. Videos of Capsaspora responding to schistosome sporocyst (Videos 1-3) or movement without schistosome present (Videos 4-6).”)

3. The Reviewer states: *‘The authors discover that FBS is also an inducer of chemokinesis in Capsaspora. Since this substance (at the same % that the authors use it) induces aggregation in Capsaspora, I wonder how the authors are able to induce separately or specifically one of the two behaviours in the cells. I have checked in materials and methods but neither there nor in the results there is an explanation in this regard. Because Capsaspora has been shown to be a model for aggregation behaviour and temporal multicellular structures, I think that would be important that this explanation is given, either in the results or in the M&M.’*

This is an important point, as indeed we have reported previously that LDLs (and HDLs) present in FBS induce aggregation. We note three important details to separate ‘aggregation induction’ from ‘chemokinesis induction’: (1) cells were plated at a low density in this experiment, where aggregation is impossible within 3 minutes, (2) cells were plated on tissue culture-treated surfaces, whereas aggregation is induced best on low-attachment surfaced, and (3) BSA was ultimately found to induce chemokinesis (but it does not induce aggregation), and LDL induces aggregation (but it is dispensable for chemokinesis). The following text was added to the Methods: “**This low cell density was chosen to avoid confounding results from cellular aggregation (which is known to occur with Capsaspora**

in the presence of FBS at higher cell densities [e.g., 8 × 10^5 cells per well] in low-attachment [non TC-treated plates]).” (lines 988-991 of marked manuscript)

4. The Reviewer states: *‘Capsaspora chemokinesis is enhanced by lysates of unrelated types of cells, even the ones from the proven host Biomphalaria, which does not seem very specific and therefore maybe unrelated to a biological role of any of these lysates into the behaviour of Capsaspora. Can the authors speculate on the nature of the response from Capsaspora cells? Maybe something that all these lysates might have in common that is enough to trigger this response?’*

We thank the reviewer and have added further discussion about the potential of the promiscuous response being ‘accidentally’ triggered by similar proteins in other organisms vs. it being a benefit to a indiscriminate predator (lines 808-824 of marked manuscript).

5. The Reviewer states: *‘Regarding the mathematical model there are some things that are not very clear to me. For example this statement “High chemo-effector levels lead to increased locomotion speed, which enhances diffusion due to random cell motion. This should not be a useful response for predators, as it would cause them to diffuse more rapidly as they approach their prey, transporting them away from their target” I understood innitially that the chemoeffector levels may difuse more due to increase cell movement, but in the second half of the sentence I understand that the cells are the ones difusing more rapidly. So, this is a bit confusing.’*

We see that the phrasing of ‘diffusion’ is confusion as it could pertain to both the chemo-effector and the cells. We have rewritten the paragraph to be explicit about when we are discussing the chemo-effector and the cells.

6. The Reviewer states: *‘Moreover a bit further down the statement “If the prey releases a large amount of chemokinesis-inducing chemicals, this would increase cell speed, and thus diffusion, encouraging higher dispersal away from the region where there is no longer prey ” seems to favour that this type of difusion would actually be advantatgeous for the predator since it would be driving away the predatory cells from those prey that were already lysated. But how this reconcilies with the observation that Capsapora rapidly prays on schistosomes if any of them has been lysated?’*

We see that our wording was confusing. We have replaced the phrase “lysed” with “devoured” in this section. The predator benefits from leaving prey after it has been fully devoured & finding new prey.

7. The Reviewer states: *‘Second statement that should be clarified :” However, coupled with irreversible attachment, the chemokinetically increased arrival rate at the schistosome implies a greater residence time for protists, which would be advantageous for predation. “ I don’t understand why the residence time increases in this circumstances, what it might increase is the number of predators arriving and attaching to the prey. Please clarify.’*

We thank the reviewer for this comment and have rewritten the paragraph to clarify that cells will reach the prey faster and therefore reside on the prey longer [lines 417-432].

8. The Reviewer states: *‘Also, I understand that these are simulations, but I am surprised by the 15 minutes max. is that number representative of what happens in the culture? 15 minutes seems very fast to me.*
For all of the above, for me it has been difficult to judge how much value and novelty the mathematical model adds to the study. Although the purpose and conclusions of the model are very well explained at the end of the introduction, I could not find in a understandable manner the proofs in the results to sustain it.’

We thank the reviewer for this comment and have repeated the simulations with new settings. We increased the time from 15 minutes to 120 minutes. Since these simulations were much longer, we used a smaller computational domain to reduce the complexity of the problem, which means that the cells began roughly twice as close to the schistosome as they did in the previous simulation. Additionally, twice as many cells were simulated, 1000 Vs 2000 and because the simulations were much longer, we changed the function that determined exudation at the surface of the schistosome. I.e., we made it so that the schistosome requires more cells present at each point to induce maximum exudation. We did this so that the domain wouldn't become completely saturated with exudate very quickly into the simulation. All of these changes are reported in the new **Supplementary Information 2**.

The increased length of the simulation, along with having more cells closer to the schistosome provided a condition that significantly benefited chemokinesis, as we hypothesized could be the case due to irreversible attachment! We therefore adjusted the entire section of the results (lines 433-749), along with the relevant parts of the abstract (lines 32-34), introduction (lines 105-107), and discussion (lines 871-891).

9. The Reviewer states: *'For Capsapora aggregation it is known that calcium is also needed to trigger this specific behaviour. I know that the authors are not exploring the internal pathways by which this chemokinetic behaviour is possible, nevertheless it would be nice if they speculate taking into account what is known for aggregation in Capsaspora or even for the case of Dyctiostelium.'*

We thank the reviewer for their suggestion and have added a new results section demonstrating that calcium signaling does not appear to be involved in chemokinesis (or aggregation) for *Capsaspora*, unlike in chemotaxis and chemokinesis of some human cells and Dictyostelium. (lines 391-405, 856-868, Methods, & Figure 4G).

10. The Reviewer states: *'I am not sure if more experiments are needed, but the conclusions are not clear to me. My background does not allow me to revise the methodological details of the mathematical model but some of the defining premises seem award to me based on what is known of Capsaspora, including what is found in this study. As I mentioned above it could be because is not well explained.'*

We have addressed the comments above (5–8) to clarify the modeling methods and conclusions.

"minor issues"

11. The Reviewer states: *'In the abstract there is a sentence where there is a "it" that should be a "its"? "Until now, little was known of how Capsaspora regulates it rate and direction"'*

We thank the reviewer for the suggestion and have completed it. (line 25 of marked manuscript)**.**

12. The Reviewer states: *'Sentence with an extra "Also": "We also have also not observed "'*

We thank the reviewer for the suggestion and have completed it. (line 376 of marked manuscript)

13. The Reviewer states: *'There are formal mistakes in the figure legends of the Videos both in the text and in Fig share (see complete report) and a few typos (also mentioned in the full report).'*

We thank the reviewer for the suggestion and have completed it.

**In addition to the changes above, we have made the requested editorial edits to the manuscript to conform to the *PLOS Pathogens* journal requirements.

Lastly, we have included a marked copy of the revised manuscript that shows changes made on revision clearly highlighted, as requested. We believe that these revisions have further strengthened the manuscript, and we are grateful to the Reviewers for their comments. We thank you in advance for your consideration of this revised manuscript.

Sincerely,

Joseph P. Gerdt, Ph.D.
Assistant Professor of Chemistry
Adjunct Professor of Biology
